# Mammalian Diving Response in Humans: Inconsistently Beneficial for Survival

**DOI:** 10.7759/cureus.108398

**Published:** 2026-05-06

**Authors:** John Hayman, Malcolm Dodd

**Affiliations:** 1 Clinical Pathology, The University of Melbourne, Melbourne, AUS; 2 Forensic Medicine, Victorian Institute of Forensic Medicine, Melbourne, AUS

**Keywords:** aortic dissection, arterial dissection, cerebral haemorrhage, essential hypertension, immersion pulmonary oedema, scuba diving, sub-arachnoid hemorrhage, sudden infant death syndrome (sids), sudden unexpected death in epilepsy, triathalon competion

## Abstract

The mammalian diving response (DR) has evolved in all air-breathing vertebrates as a means of life preservation when immersion in water precludes normal respiration. It is highly developed in diving mammals but present in all Mammalia, including man, as well as other vertebrates. In man, the response consists of apnoea, bradycardia, peripheral and splanchnic vessel constriction with increased venous return to the heart, and increased systolic and pulse pressures.

Components of this response may contribute to unexpected deaths in infants and to deaths associated with epileptic seizures. Although direct evidence is lacking, the response provides an explanation for immersion pulmonary oedema in swimmers and divers and for the occasional dissecting aneurysm and other vascular catastrophes that have been reported in scuba divers. Persistence of a vigorous dive response beyond infancy and into adulthood may be a prelude to the development of essential hypertension in later life.

Although causation remains unproven, the DR provides a physiologically plausible unifying mechanism for a range of otherwise disparate adverse clinical events. Prospective clinical monitoring in at-risk populations by annual blood pressure recordings and testing of pulse response to facial wetting, together with minor changes in competitor practices, may clarify the role of the DR and guide preventive strategies.

## Introduction and background

**Figure 1:**
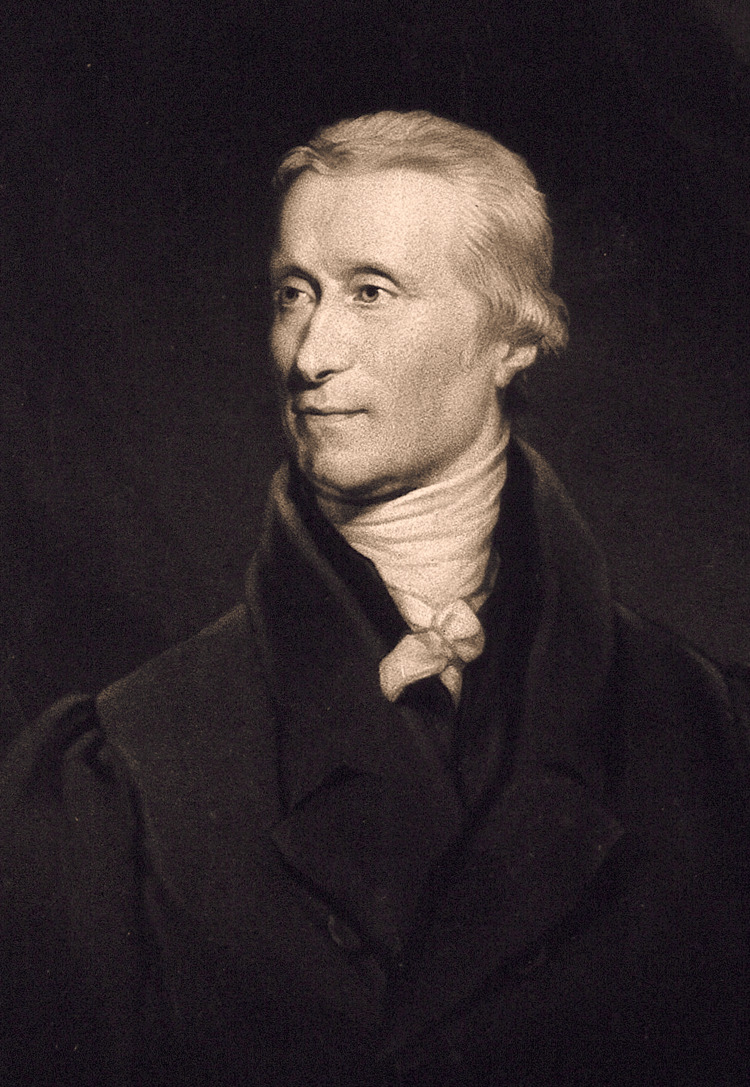
Edmund Goodwyn, MD (1756-1829) As well as describing the dive response, Goodwyn made important contributions to medicine, including demonstrating that death from drowning was not due directly to water entering the lungs but rather to preventing air entry. He proposed artificial ventilation as a means of resuscitation rather than other proposed means such as heat, electricity, and bleeding. Source: From Wikimedia Commons, original portrait by the National Portrait Gallery. Licensed under CC BY 4.0.

The mammalian diving response (DR) is a complex autonomic reflex occurring in all mammals, including man. It is not a recent discovery. Edmund Goodwyn (1756-1829) is credited with the first description of the dive response in his MD graduation thesis (Figure 1), submitted to the University of Edinburgh in 1786 [1]. It also occurs in other vertebrates, including birds; hence the preferred acronym DR. It is most developed in diving mammals and has been studied extensively in phocid seals. Laboratory studies have also been carried out in trained laboratory rats [2]. 

**Figure 2:**
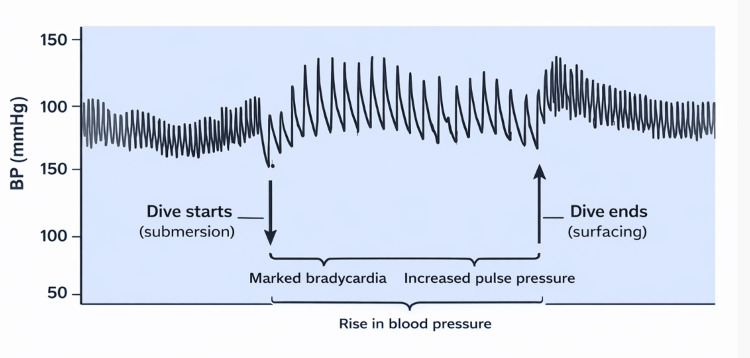
Graph showing the effects of submersion in a rat trained to dive and swim through an underwater channel Down arrow - dive commences; up arrow - dive ends. Note the marked bradycardia commencing immediately on submersion, with an increase in pulse pressure followed shortly by a rise in blood pressure. Resting systolic BP: 130 mmHg; submerged: 150 mmHg; corresponding pulse pressures: 20 mmHg and 40 mmHg. Source: Adapted from Panneton [2], with permission for adaptation obtained.

The DR consists of apnoea, bradycardia, and hypertension with increased pulse pressure (Figure 2), along with selective vasoconstriction, the latter affecting splanchnic and peripheral vessels and resulting in increased venous return to the right heart. Apnoea may serve to initiate the response, as well as being part of the response itself [2].

The overall effect of the response is to conserve intrinsic oxygen supply, with the blood supply to the brain and heart being maintained at the expense of tissues more tolerant of hypoxia. As a sequel, blood flow with an increased pulse pressure occurs in the aorta and in the coronary, cervical, and intracranial arteries, but not in those forming the blood supply of peripheral and splanchnic tissues. The vasoconstriction of selected vessels serves to reduce the systemic lactic acidosis that would otherwise occur with the return of blood from hypoxic areas. This peripheral and splanchnic vasoconstriction results in an increased venous return to the right heart and increased cardiac output from the right ventricle into the pulmonary circulation.

In the rat, an animal adapted to underwater swimming, the response exists throughout its life, with marked bradycardia on immersion (Figure 2). In humans, the response is present in all infants; in these children, the response may be initiated not only by facial wetting but also by simple breathing on the face [3]. In adults, the response remains, although it is generally less reactive [4]. Bradycardia, however, has been shown to occur in all participants in a recent small study of experienced divers [5].

In humans, the initiation of the response occurs upon contact with cold water on the face, with stimulation occurring via the ophthalmic branch of the trigeminal nerve. A study of different facial areas in eliciting the bradycardia component of the DR demonstrated that the forehead was the most sensitive area [6].

Diving mammals have evolved anatomical structures that serve to reduce the harmful effects of the DR. A balloon-shaped, elastic, bulbous aorta has evolved in seals (Pinnipedia and Cetacea), which serves to lessen pulse pressure and to maintain blood flow to the brain and heart during the extreme bradycardia that occurs in these aquatic mammals [7]. Cetaceans and other seals have developed vena caval sphincters and venous retes to control venous return to the right heart [8]. The human mammal, possessing a DR but lacking these anatomical adaptations, may suffer adverse effects in an aquatic environment. In addition, adverse events attributable to the DR may occur far from water.

Although of possible survival benefit to the foetus during the birth process and to man when in an aqueous environment [9], the DR may have disadvantageous, occasionally fatal effects in a variety of circumstances [10]. Differing components of the DR may contribute to these detrimental outcomes in a variety of situations.

This is a hypothesis-driven review proposing how different components of the DR may be implicated in different adverse events in humans. The role of the DR in the pathogenesis of these conditions remains contentious, but the mechanisms are physiologically plausible. Apnoea may contribute to sudden infant death syndrome (SIDS) and sudden unexpected death in epilepsy (SUDEP); peripheral vasoconstriction with increased venous return to the right heart may contribute to immersion pulmonary oedema (IPE); and bradycardia with high systolic and pulse pressures may contribute to dissecting aneurysms of cervical and cerebral vessels, occasional cerebral and subarachnoid haemorrhage, and dissecting aortic aneurysms. A vigorous DR persisting into adulthood may be the forerunner of established hypertension in later life. These adverse events are examined in greater detail.

## Review

Materials and methods

A search was made of the medical literature using PubMed and Google Scholar for articles implicating and discussing the role of the DR in the pathogenesis of SIDS and SUDEP. Interest in these conditions was prompted by our knowledge and experience with the DR in fatal diving accidents [10]. The search was widened to include events in swimmers and divers that may have been precipitated by this response, particularly focusing on reported occurrences of IPE and dissecting aneurysms.

A possible association with later development of persistent hypertension was a fortuitous discovery from one reference [11], which prompted further search.

Results

The DR has been implicated in SIDS, in SUDEP, and in IPE [9]. To this may be added dissecting aneurysms of the aorta, cervical, and cerebral arteries, events reported in scuba divers [10,12]. Dissecting aneurysms are largely confined to scuba divers, although one case has been reported in a 50-year-old hypertensive male triathlete who was using intermittent apnoeic (pyramid) breathing in the swimming phase of the competition [13]. Two cases of fatal aortic dissection associated with swimming have been reported separately in boys aged 11 and 12 [14,15].

As well as dissection, with the increased pulse and systolic pressures in cerebral and coronary arteries, the DR may be a precipitating cause of some cases of intracerebral [16], subarachnoid aneurysmal [17], and non-aneurysmal haemorrhage [18]. Although rare, these are events that have been reported after ice-water dousing [19], with scuba and breath-hold divers, as well as with surface swimmers.

One case only of dissecting aneurysm of a coronary artery has been reported [20]. Cardiac events, however, are not unusual in scuba divers. It is thought that coronary artery dissection may occur more frequently but is not recognised as such, being misdiagnosed both on angiography and at autopsy as coronary occlusion due to haemorrhage into a plaque, which may also occur [21].

Added to these, a final conjecture is that an active DR persisting into adulthood, with increased blood pressure and pulse pressures, may be the forerunner of continued hypertension [11,22].

Evidence as to the causality of the DR in any of these events should be seen as suggestive, plausible, but inconclusive. The relationship to the DR may be supported by future guided monitoring. Each of these adverse clinical events will now be examined in some detail, along with evidence linking them to the DR.

Sudden Infant Death Syndrome (SIDS)

SIDS is described as the unexpected death of an infant less than one year of age, with the onset of the fatal episode apparently occurring during sleep-a death that remains unexplained after a thorough investigation [23]. Many points of view have been put forward regarding “the cause” of SIDS, but no single cause has been established [24]. This tragic event should be regarded as a syndrome rather than a specific disease entity, with a variety of causes or contributing factors.

Among these proposed causes is the activation of the DR, with high probability among infants with a vigorous response and possible genetic background [25]. Involvement of the DR in SIDS has been advanced by several authors and is supported by a reduction in mortality following advice against infants sleeping prone [26]. With the face down, there is a greater possibility of facial wetting from mucous secretions, with DR stimulation from the infant’s own respiration. The pulmonary congestion and oedema seen at autopsy in SIDS cases could be the sequel to the increased return of blood following the peripheral vasoconstriction that occurs with the DR, and the petechial haemorrhages seen on serous surfaces are consistent with apnoea and subsequent anoxia [27].

However, SIDS still occurs in infants sleeping supine; the DR is only one of several possible stressors.

The brainstem gliosis found at post-mortem in these infants may be evidence of hypoxia prior to the fatal event [28]. Other evidence of prior hypoxic events includes retained extramedullary haemopoiesis, larger residual brown fat deposits, and increased smooth muscle in small pulmonary arteries. The arterial changes may be a response to alveolar hypoxia associated with episodic apnoea [29]. The chromaffin cell hyperplasia found in adrenal glands is evidence of increased catecholamine secretion, which in turn may be a response to hypoxia and CO₂ retention subsequent to hypoventilation. A suggestion that may be added is that increased catecholamine secretion is a physiological response to DR-induced episodes of bradycardia.

For the present, SIDS should be regarded as a tragic happening at a susceptible period of a child’s life. Rather than a precise diagnosis, the syndrome should be seen as having a multifactorial causation, including the DR, and associated with recurrent prior hypoxic stress.

Sudden Unexpected Death in Epilepsy (SUDEP)

Patients with epilepsy have a markedly increased mortality, with non-accidental deaths occurring approximately 20 times more frequently than in the general population [30]. Specific findings at post-mortem are often absent. In one analysis of 31 autopsied cases, the mechanism of death was attributed to cardiopulmonary failure in about half, and to asphyxia in a further quarter [31]. The immediate cause of death was classified as SUDEP or epileptic state in 42% of cases, while the remainder were attributed to a variety of unrelated conditions.

One proposed mechanism is that prolonged apnoeic generalised tonic-clonic seizures (GTCS) trigger an exaggerated form of the DR [32]. Support for this hypothesis comes from observations made in epilepsy monitoring units, where autonomic and respiratory dysfunction have been documented preceding SUDEP. A comprehensive assessment of cardiorespiratory arrests during monitored seizures demonstrated severe disturbances of cardiac and respiratory function in the post-ictal period following GTCS that culminated in SUDEP [33]. Transient interruptions of breathing preceded terminal apnoea and ultimately terminal asystole. A recent paper detailing dissecting aneurysms occurring in association with epileptic attacks provides strong evidence that the DR is activated during these episodes [34]. Neuroimaging studies have also identified structural abnormalities in cortical, subcortical, and brainstem regions in patients who subsequently died of SUDEP, as well as in those considered at greatest risk.

A reasonable interpretation is that the DR acts in the setting of an already compromised autonomic and respiratory control system - one that is unable to respond adequately to the profound physiological disturbances induced by seizures.

Activation of the DR also provides a plausible explanation for some of the post-mortem findings. Increased venous return to the heart could contribute to the pulmonary oedema and haemorrhage frequently observed in SUDEP. In addition, simultaneous sympathetic and parasympathetic cardiac stimulation may promote fatal cardiac arrhythmias.

Immersion Pulmonary Oedema (IPE)

IPE is a relatively common condition affecting surface swimmers as well as breath-hold and scuba divers [35]. It typically occurs in young, healthy, athletic individuals and appears to be more common in females [36]. Mild symptoms are frequent and include cough and dyspnoea with frothy sputum, occasionally accompanied by haemoptysis, developing during or shortly after surface swimming or shallow immersion [37]. In more severe cases, marked breathlessness may occur, occasionally progressing to collapse, with loss of consciousness and, rarely, death from respiratory failure. Because the lungs are fluid-filled, the condition may be confused with, and misdiagnosed as, drowning at autopsy.

Athletic swimmers, including triathlon competitors and military trainees, may be at increased risk. Female athletes appear to be more susceptible than their male counterparts [36]. Paradoxically, high levels of training and physical fitness may increase, rather than decrease, the risk. One explanation is that increased muscle mass augments venous return to the heart during immersion as a consequence of peripheral vasoconstriction. The increased risk observed in military recruits may relate to the combination of high muscle mass, strenuous exertion, and cold-water exposure.

During immersion with exercise, the mammalian DR is thought to be activated. Peripheral and splanchnic vasoconstriction associated with the DR increases venous return to the right heart, resulting in increased cardiac output to the pulmonary circulation. Swimmers who have experienced IPE demonstrate higher pulmonary artery pressures and pulmonary artery wedge pressures compared with incident-free controls [38]. Sildenafil, a drug which reduces pulmonary hypertension, has been shown to reduce the risk of IPE. The elevation of pulmonary artery and pulmonary capillary pressures provides a plausible mechanism for the development of pulmonary oedema and suggests a potential role for the DR in the pathogenesis of IPE.

Dissecting Aneurysm: Aorta, Cervical, and Cerebral Arteries; Coronary Artery; Cerebral and Subarachnoid Haemorrhage

Dissecting aneurysms of the aorta, cervical, and intracranial cerebral arteries have been reported in scuba divers, and there has been one report of dissection of a coronary artery with subsequent myocardial infarction [10,12,20]. Aneurysmal and non-aneurysmal subarachnoid haemorrhage and intracerebral haemorrhage have also been reported during scuba diving [17,18].

**Figure 3:**
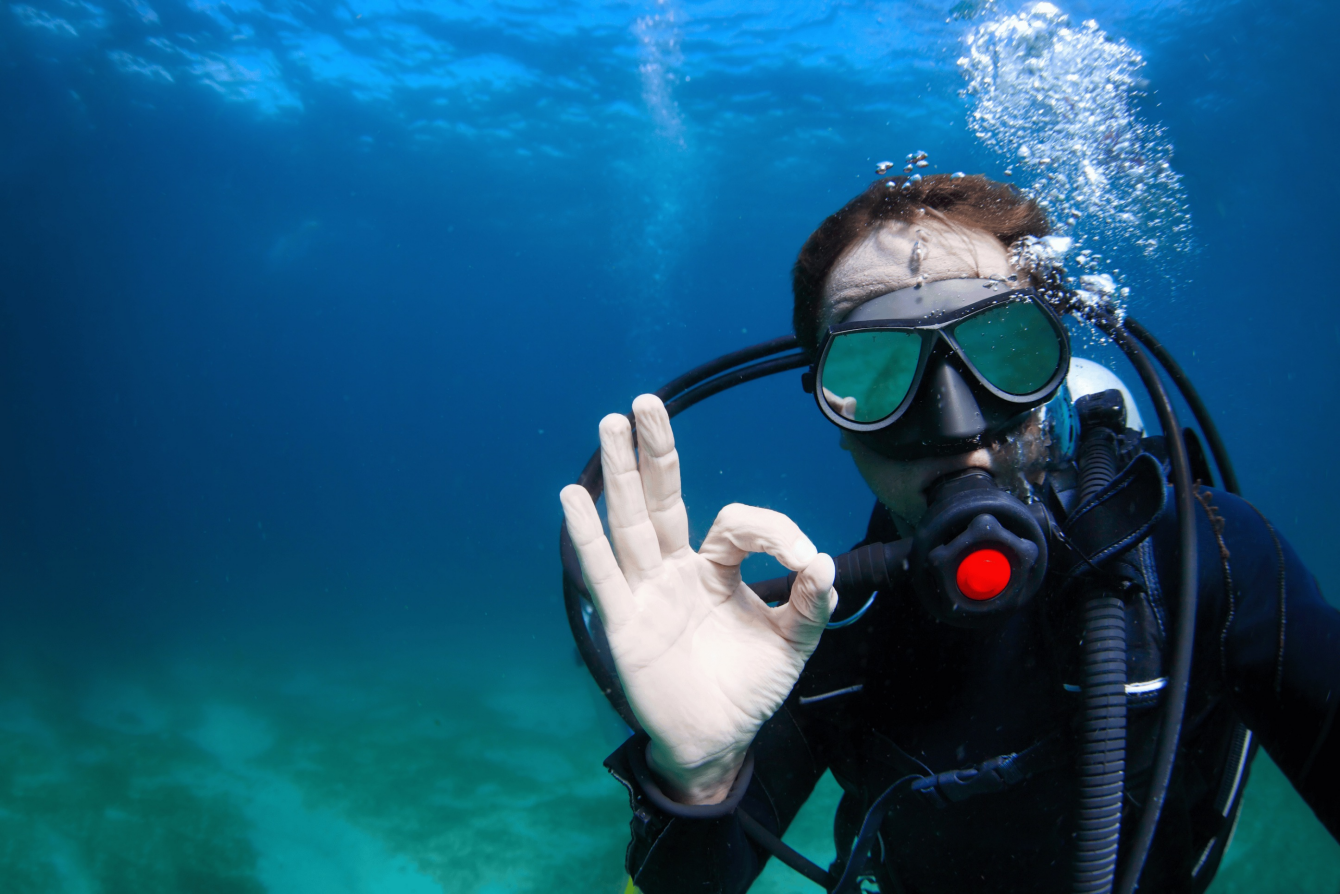
Image of a scuba diver giving the “OK” sign; note how the forehead is exposed Source: Image licensed from Shutterstock (Shutterstock, Inc., New York, NY, USA).

Although in scuba diving the face is partly covered by a mask, often with a hood, the face may still become wet. Clearing a water-filled mask is part of scuba dive training (Figure 3). Furthermore, wetting of the forehead alone is sufficient to activate the DR [5], and forehead wetting occurs even if mask and hood are worn. The DR has been shown to be activated with scuba diving [4].

Adverse cardiovascular events during surface swimming are less frequent. Two cases of fatal dissecting aortic aneurysm have been reported in surface swimmers, boys aged 11 and 12 years [14,15]. A hypertensive male aged 52 developed a dissecting aneurysm in his left internal carotid artery when practising intermittent apnoeic swimming [13]. This resolved with conservative treatment. He also had a small aneurysm on his right internal carotid artery, which was interpreted by the radiologist as a sequel to a prior dissection on that side. As well as swimming, there is an isolated case attributed to the DR of cerebral haemorrhage after dousing with ice-cold water [19].

Hypertension

Evidence for the role of the DR as a prelude to established hypertension is slender and circumstantial. It is, however, a proposal worth considering. There is the report by Wilmshurst et al. of hypertension developing after cold-induced IPE, postulated in this paper to be a sequel to the DR [12]. Episodic hypertension, which is a feature of the DR in humans, is recognised as a precursor to fixed or essential hypertension [22].

Discussion

This is not intended as a comprehensive review - either of the DR itself or of the various conditions discussed. Rather, it focuses on an important and often overlooked topic, highlighting how different aspects of the DR may contribute to a range of adverse outcomes. The DR can produce relatively minor symptoms, but in some cases, it may lead to severe, even fatal consequences. The term “diving response” may, in fact, be something of a misnomer, as its adverse effects are not confined to aquatic environments and may also occur in non-aqueous settings.

IPE occurs in both surface swimmers and divers, and episodes with minor symptoms are relatively common. Susceptibility in athletes has been attributed to increased venous return associated with greater muscle mass, while the higher incidence in females has been ascribed to a relatively smaller pulmonary vascular bed available to accommodate this increased venous return. Mild episodes may represent a prelude to more severe events. Preventative measures may include avoidance of fluid overload prior to exertion and, in selected individuals, the use of a diuretic or sildenafil [38].

Dissecting aneurysms appear to be largely confined to scuba divers: 19 cases have been reported in this group compared with only three among the much larger population of swimmers [10]. Notably, two of the three swimmers, both with aortic dissection, were boys aged 11 and 12 years, who would be expected to exhibit a more vigorous DR [14,15]. The third case, with internal carotid dissection, involved a 50-year-old man practising intermittent apnoeic swimming [13]. Apnoea, a recognised component of the DR, is also a known precipitating factor [2]. In divers, haemodynamic forces related to increased ambient pressure at depth - absent in surface swimmers - are likely to augment the DR and contribute to vascular stress.

These hypotheses are amenable to testing. Non-invasive devices capable of recording pulse rate, oxygen saturation, and blood pressure are now widely available and could be deployed in at-risk situations. The onset of the DR may be identified by characteristic bradycardia.

Well-conducted scuba training programmes typically require medical assessment prior to participation; similar screening should be considered for endurance swimmers and triathletes. A simple test for a persistent or exaggerated DR involves applying a cold, moist cloth to the forehead while monitoring heart rate and blood pressure responses. Although long-term follow-up of participants is challenging, it is not impossible. Pending definitive evidence, pragmatic measures may reduce risk: infants should be placed to sleep supine in a smoke-free environment; overhydration prior to endurance events in water should be avoided; and intermittent apnoeic breathing during exertion should be discouraged. Competitors with recurrent episodes of IPE may benefit from prophylactic pharmacological therapy [39].

## Conclusions

Adult individuals with an enhanced DR are identifiable by non-invasive testing and may be at increased risk of DR-related adverse events. Identified susceptible individuals may be monitored for adverse event occurrence. These, together with a supposedly low-risk control group, will provide evidence supporting or refuting these proposed hypotheses. Minor changes in practice and behaviour can reduce the occurrence of these adverse events. Prophylactic drug therapy may be used to reduce risk when there is a high probability of an adverse event occurring. The DR should be considered as a possible underlying mechanism in any unexplained drowning, cardiac, or neurological event occurring in relation to swimming or diving. IPE may be potentially life-threatening. Those who have suffered a prior episode are advised to abort competition in any endurance swimming or triathlon event at the first appearance of symptoms. Risk may be reduced by avoiding overhydration before an event and by taking prophylactic medication.

More specifically, patients suffering from epilepsy with frequent GTCS should have cardiac, respiratory, and blood oxygen monitoring until normal breathing is restored. Dousing with ice-cold water is not a risk-free challenge. Intermittent apnoeic breathing during any swimming event should be avoided.
